# Combination of double negative T cells and anti-thymocyte serum reverses type 1 diabetes in NOD mice

**DOI:** 10.1186/s12967-016-0815-y

**Published:** 2016-02-24

**Authors:** Tianhui Liu, Min Cong, Guangyong Sun, Ping Wang, Yue Tian, Wen Shi, Xinmin Li, Hong You, Dong Zhang

**Affiliations:** Research Center, Beijing Friendship Hospital, Capital Medical University, 95 Yong-an Road, Xi-Cheng District, Beijing, 100050 China; Beijing Key Laboratory of Tolerance Induction and Organ Protection in Transplantation, Beijing, China; Beijing Key Laboratory of Translational Medicine in Liver Cirrhosis & National Clinical Research Center of Digestive Diseases, Liver Research Center, Beijing Friendship Hospital, Capital Medical University, Beijing, China

**Keywords:** Cell therapy, CD4^−^CD8^−^ regulatory T cells, Anti-thymocyte serum, NOD mouse, Type 1 diabetes

## Abstract

**Background:**

Double-negative (DN) T cells could delay the onset and the progression of autoimmune diabetes, yet they were less efficient on reversing autoimmune diabetes. The aim of this study was to investigate whether the combination of DN T cells and anti-thymocyte serum (ATS) could reverse new-onset diabetes in NOD mice.

**Methods:**

The regulation of different subsets of T cells in vitro and in vivo by ATS and DN T cells were examined using flow cytometry. At the day of diabetes onset, ATS was administered on the same day and 2 days later, and DN T cells were transferred at day 7. The reversion of diabetes was assessed by monitoring blood glucose levels.

**Results:**

The efficacy of inhibition of DN T cells on CD8^+^ T cells was lower than that on CD4^+^ T cells both in vitro and in vivo. ATS resulted in a significant depletion of CD8^+^ T cells, while DN T cells were less sensitive to ATS depletion. 80 % diabetic NOD mice achieved long term (6 months) reversion of diabetes by combined ATS and DN T cells treatment, compared to 16 % in ATS single treatment and none in DN T cell single treatment. DN T cells preferentially resided in spleen and pancreatic draining lymph nodes in ATS plus DN T cells treated NOD mice.

**Conclusions:**

DN T cells plus ATS therapy show promising reversion effects on diabetic NOD mice due to a shift of balance from a destructive T cell response to one that favors DN T cell regulation.

## Background

Type 1 diabetes is an autoimmune disease in which the patient fails to develop or loses tolerance to self-antigens at a young age. Consequently, auto-aggressive T cells gradually infiltrate the pancreas and selectively destroy the insulin-producing beta cells, eventually leading to overt diabetes [1, 2]. Both CD4^+^ and CD8^+^ cytotoxic T lymphocytes contribute to beta cell killing [3–5]. One mechanism that may lead to the development of type 1 diabetes is the defective development, activation or function of regulatory T cells [6–8]. Several subsets of regulatory T cells have the ability to suppress the proliferation and function of autoreactive T cells and prevent type 1 diabetes development, including naturally occurring and induced populations of CD4^+^CD25^+^FoxP3^+^ T cells, CD8^+^ treg cells, NK T cells and CD4^−^CD8^−^ (double-negative, DN) T cells [9–13]. While currently, it is still a great challenge to reverse new diabetes successfully using adoptive cell therapy.

The DN T cells that are capable of downregulating the immune response comprise only 1–5 % of αβ T cell receptor (TCR)+ T cells in the peripheral lymphoid tissue of normal mice and humans [14]. DN T cells also play a homeostatic role in autoimmune diabetes. Diabetes-prone mice carry fewer DN T cells and this contributes to the increased susceptibility of these mice to developing the disease [15]. Moreover, peptide-activated antigen-specific transgenic DN T cells can prevent autoimmune type 1 diabetes development [12]. DNCD3 splenic T cells from young non-obese diabetic (NOD) mice were demonstrated to be able to induce long-lasting protection against diabetes subjected to an adoptive cell transfer protocol [16].

We identified a new differentiation pathway, by which a subset of proliferated CD4^+^ T cells converted to DN T cells after antigen-triggered or homeostatic proliferation in vitro and in vivo. Using this novel pathway, tens of millions of DN T cells can be rapidly produced [17]. We also reported that CD4^+^ T cells converted to pancreatic islet beta cell antigen-specific DN T cells can prevent the development of autoimmune diabetes and promote islet allograft survival in NOD mice [13]. Because DN T cells proliferated at very low levels in vitro and in vivo [17] and DN T cell inhibition on target cells was shown to be dependent on cell-cell contact [18], lymphodepletion treatment to diminish pathogenic T cells population prior to DN T cell therapy might be an effective method to achieve more profound regulation.

In this study, we investigated the regulation of different subsets of T cells in vivo and in vitro by anti-thymocyte serum (ATS) and DN T cells. We also report the use of CD4^+^ T cell converted antigen-specific DN T cells in combination with ATS to suppress and reverse autoimmune diabetes in NOD mice with newly developed type 1 diabetes.

## Methods

### Animals

Male C57BL/6 (H2^*b*^), C57BL/6 RAG^−/−^ (H2^b^), C57BL/6 congenic for CD45.1 (H2^*b*^), DBA/2 (H2^*d*^) and female NOD (H2^g^) were purchased from Vital River Laboratories (Beijing, China) and the Jackson Laboratory (Bar Harbor, ME, USA). All mice were maintained in pathogen-free facilities at Beijing Friendship Hospital. All protocols were approved by the Institutional Animal Care and Ethics Committee.

### Purification of CD4^+^ T cells, CD8^+^ T cells

Single-cell suspensions of mouse spleens and lymph nodes were prepared. CD4^+^ T cells were isolated by T cell enrichment column (R and D Systems, Minneapolis, MN, USA) followed by Ter119, B220, CD8, CD11b, TCR γδ, CD25 and NK1.1 positive cell depletion. CD8^+^ T cells were isolated by T cell enrichment column followed by Ter119, B220, CD4, CD11b, TCR γδ and NK1.1 positive cell depletion.

### Conversion of DN T cells in vitro

Conversion of DN T cells in vitro was performed as previously described [13]. Briefly, mature dendritic cells (mDCs) were harvested from lipopolysaccharide-stimulated bone marrow cells of DBA/2 or NOD mice, and separated by CD86-positive selection. C57BL/6 CD4^+^ T cells were incubated with DBA/2 mDCs with 50 ng/ml rmIL-2 (Peprotech, Rocky Hill, NJ, USA). CD4^+^ T cells from NOD mice were cultured with NOD mDCs in the same conditions described above plus the addition of 1 µg/ml GAD65 peptides. CD3^+^CD4^−^CD8^−^ DN fractions were isolated from mixed lymphocyte reaction (MLR) and sorted using a FACS (AriaII; BD Biosciences, San Diego, CA, USA).

### In vitro suppression assays

Carboxyfluorescein diacetate succinimidyl ester (CFSE; Molecular Probes, Eugene, OR, USA) labeled CD4^+^ or CD8^+^ T cells (1 × 10^5^/well) from CD45.1 congenic C57BL/6 mice were co-cultured with DBA/2 mDCs (0.25 × 10^5^/well) for 4 days. The same amount of converted B6 DN T cells were added to MLR as regulatory cells. CD4^+^ and CD8^+^ T cell proliferation was examined by flow cytometry.

### ATS treatment in vitro

For ATS treatment of mouse splenocytes, freshly isolated cells were cultured in complete medium with 2 µl/ml of either ATS or rabbit serum (Accurate Chemical and Scientific Corporation, Westbury, NY, USA). Twenty-four hours following culture initiation, splenocytes were harvested and TCR-β^+^, CD4^+^, CD8^+^ T and DN T cells were examined by flow cytometry.

### ATS treatment in vivo

Eight-week-old NOD mice were treated with two intraperitoneal injections of ATS or rabbit serum (50 µl/mouse) at day 0 and 2. Mice were monitored continuously by examining TCR-β^+^, CD4^+^, CD8^+^ T and DN T cells in peripheral blood monocytes (PBMCs) using flow cytometry.

### Adoptive transfer and skin transplantation

C57BL/6 DN T cells (1 × 10^5^) in combination with 1 × 10^5^ naive CD4^+^CD25^−^ T cells or CD8^+^ T cells were transferred to C57BL/6 RAG^−/−^ mice by tail vein injection. On the same day, full thickness 1 cm^2^ tail skin grafts from DBA/2 mice were transplanted to the C57BL/6 RAG^−/−^ mice. Graft survival was monitored by daily visual inspection. Graft rejection was defined as complete necrosis and loss of viable skin tissue.

### Adoptive transfer and diabetes reversion

Mice were monitored for diabetes development by measuring blood glucose levels twice weekly. Diabetes onset was defined as blood glucose levels of >12 mmol/L in two consecutive measurements. In the event of new-onset diabetes, two doses of 50 µl ATS were given to diabetic NOD mice on day 0 and 2 by intraperitoneal injection. On day 7, ex vivo converted GAD65 primed DN T cells (1 × 10^6^) were transferred to ATS-treated mice by tail vein injection. Blood glucose levels of treated mice were inspected every other day.

### Flow cytometric analysis

Cultured cells or PBMCs from treated mice were harvested at various time points and analyzed for proliferation or the expression of various cell surface markers. All samples were analyzed on an Aria II flow cytometer (BD Biosciences). Data was analyzed using FlowJo software (Treestar; FlowJo, Ashland, OR, USA).

### Histologic examination

Pancreas were isolated 4 weeks after diabetes onset, and were fixed in 4 % paraformaldehyde, paraffin embedded, and sectioned. H and E staining was performed.

### Statistical analysis

Analyses for statistically significant difference were performed using the Student’s *t* test and one-way ANOVA test. The effects of DN T cells on diabetes reversion in the adoptive transferred models and the skin transplant model were statistically analyzed using a log-rank test. *p* values <0.05 were considered significant.

## Results

### CD4^+^ T cells converted DN T cells showed strong immune regulation on CD4^+^ T cells, but less suppression on CD8^+^ T cells both in vitro and in vivo

**Fig. 1:**
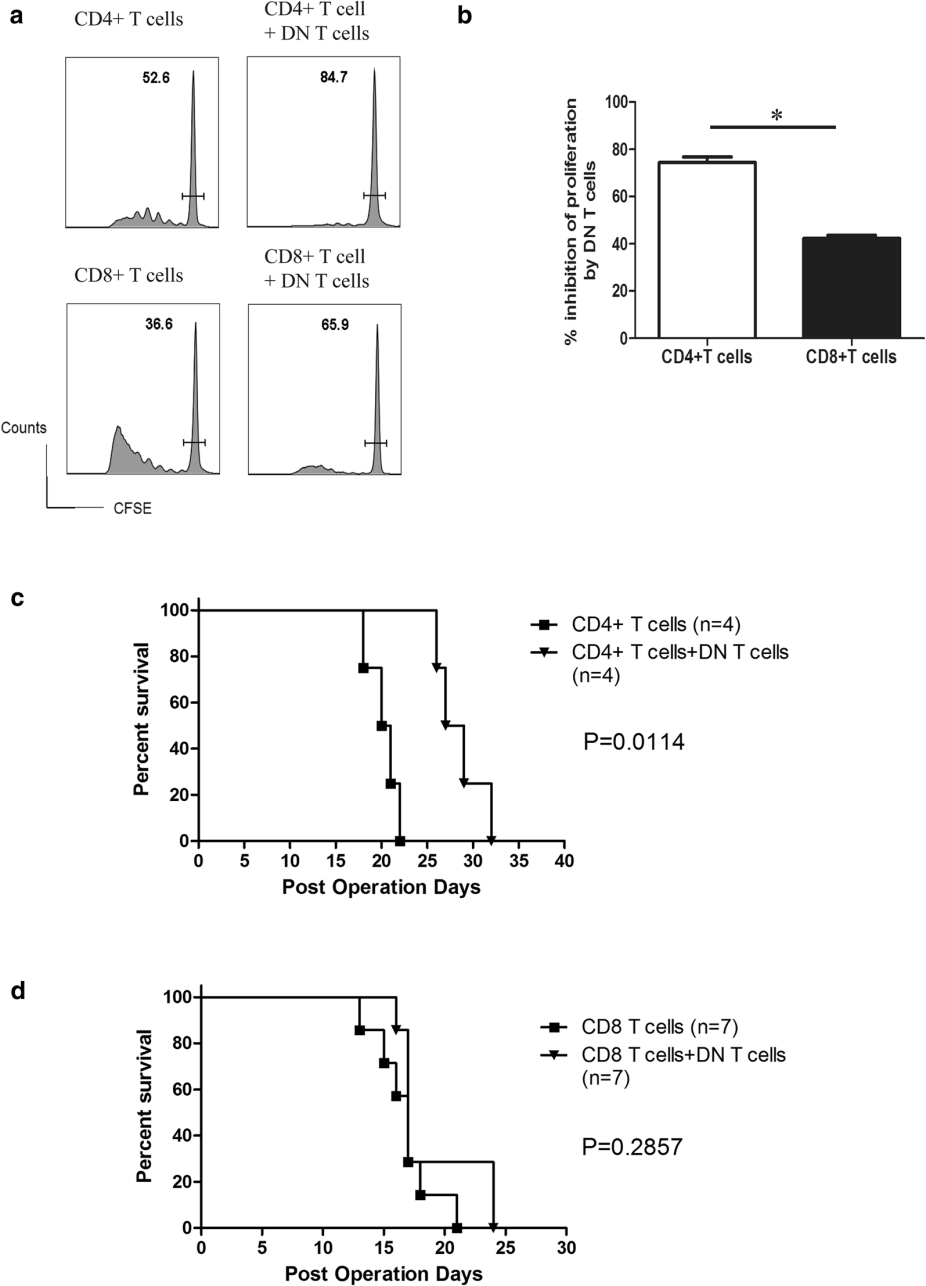
DN T cells showed different immune regulation of CD4^+^ and CD8^+^ T cells in vitro and in vivo. **a** DN T cells potently suppressed CFSE-labeled CD4^+^ and CD8^+^ T cell proliferation triggered by mDCs in vitro. The *horizontal bars* gate the un-dividing cells, and the numbers refer to the percentages these cells comprise of the total CD4^+^ or CD8^+^ T cells respectively. **b** The data are shown as percent inhibition of proliferation compared with controls, to which no DN T cells were added. The results reported are representative of three experiments with similar results. **c** The rejection of a skin graft from DBA/2 mice transplanted to C57BL/6 RAG^−/−^ mice was induced by adoptive transfer of naïve C57BL/6 CD4^+^CD25^−^ T cells or CD8^+^ T cells. C57BL/6 DN T cells were co-transferred by tail vein injection. Graft survival was observed by daily visual inspection. DN T cells suppressed naïve CD4^+^CD25^−^ T cell-triggered skin allograft rejection. **d** DN T cells failed to prolong naïve CD8^+^ T cell-triggered skin allograft rejection. Statistical analysis was performed using a log-rank test

As shown in Fig. 1a, C57BL/6 DN T cells that were incubated with mature DBA/2 mDCs in vitro potently suppressed C57BL/6 (CD45.1) CD4^+^ and CD8^+^ T cell proliferation triggered by the same alloantigens (DBA/2 DCs) in vitro. The inhibition efficacy of DN T cells on CD8^+^ T cells (46.2 %) was lower than that on CD4^+^ T cells (67.7 %) (Fig. 1b). The differences were more profound in vivo. Compared with control, significant prolongation of skin allograft survival on RAG^−/−^ recipients occurred when equal numbers of DN T cells and CD4^+^CD25^−^ T cells were co-transferred (Fig. 1c; mean graft survival time of 28 days vs 20.5 days; *p* = 0.0114). In contrast, DN T cells did not protect the skin graft rejection triggered by CD8^+^ T cells (Fig. 1d; *p* = 0.2857).

### ATS treatment preferentially depleted CD8^+^ T cells while DN T cells were resistant to ATS both in vitro and in vivo

**Fig. 2:**
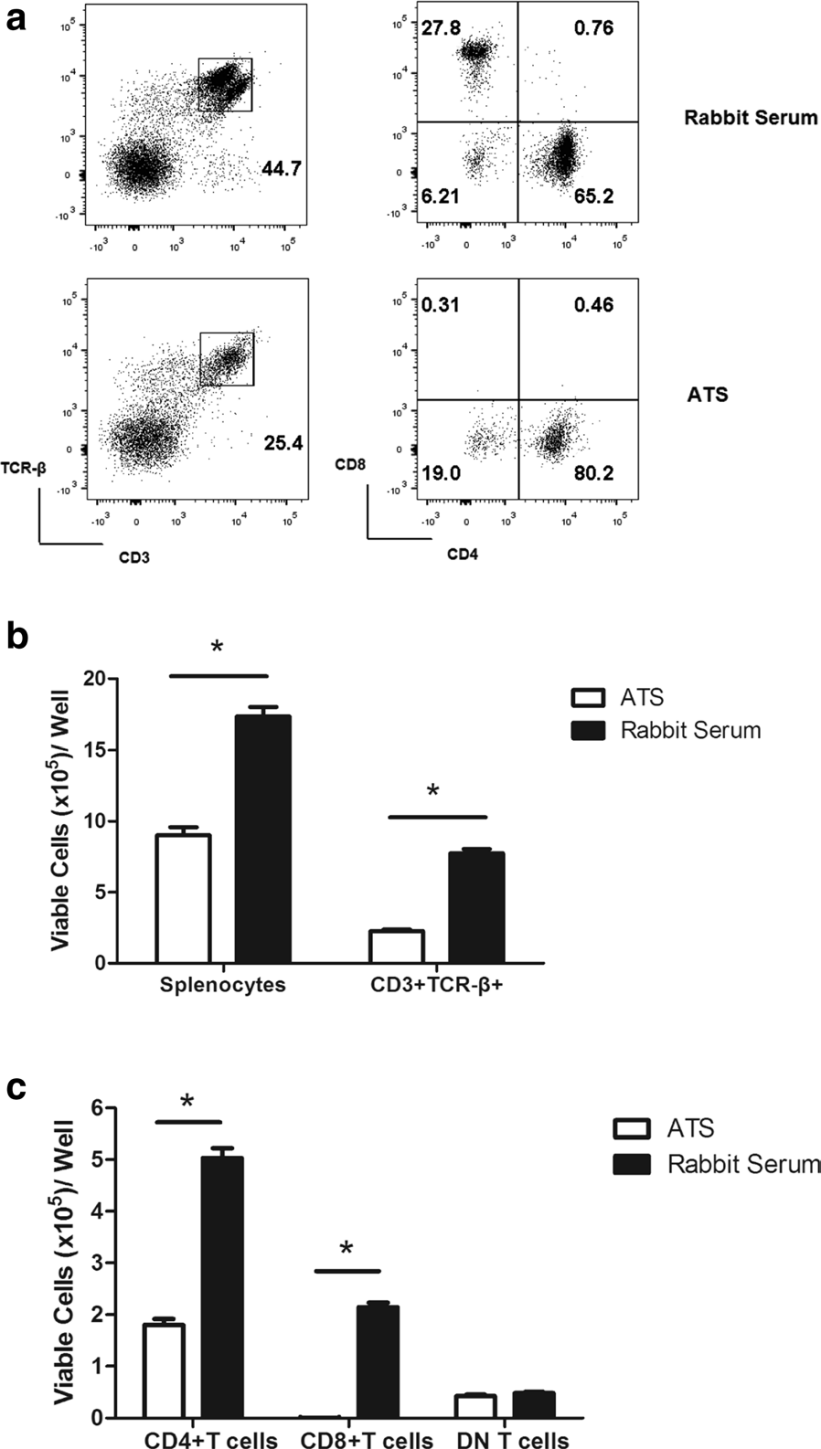
ATS treatment differentially depletes T cells from spleen after 24 h in vitro. C57BL/6 splenocytes were cultured with 2 µl/ml ATS or rabbit serum and **a** the percentage of TCR-β^+^, CD4^+^, CD8^+^ and DN T cells were evaluated 24 h later by flow cytometry. The numbers in the *left panels* refer to the percentages of CD3^+^TCR-β^+^ cells in the total lymphocyte pool, the numbers in the *right panels* refer to the percentages of CD4^+^,CD8^+^ and DN T cells among the CD3^+^TCR-β^+^ lymphocytes. Data are representative of three experiments performed with similar results. **b** Absolute numbers of splenocytes and CD3^+^TCR-β^+^ T cells 24 h following the initiation of the cultures. **c** Absolute numbers of CD4^+^, CD8^+^ and DN T cells 24 h following the initiation of the cultures. Data shown are the means (±standard deviation [SD]) of three separate experiments

Both anti-thymocyte globulin (ATG) and ATS therapy can largely eliminate T cells from peripheral blood. It is debated whether ATG therapy preferentially depletes certain subsets of T cells. For instance, Xia et al. [19] have reported that ATG depletes CD8^+^ T cells more efficiently than CD4^+^ T cells in both peripheral blood and lymphoid organs. We investigated changes of the absolute numbers and percentages of different T cell subsets in vitro. As shown in Fig. 2a, the percentage of CD3^+^TCR-β^+^ cells in splenocytes decreased from 44.7 to 25.4 % with ATS treatment, and the absolute number of CD3^+^TCR-β^+^ cells also decreased significantly (Fig. 2b). The relative percentage of CD4^+^ T cells among the CD3^+^TCR-β^+^ lymphocytes changed from 65.2 to 80.2 %, while CD8^+^ T cells (27.8–0.31 %) was almost eliminated by ATS treatment (Fig. 2a). Both absolute number of CD4^+^ and CD8^+^ T cells decreased, compared to CD4^+^ T cells, the absolute number of CD8^+^ T cells was more significantly decreased post-ATS treatment (Fig. 2c). Compared to the rabbit serum group, among all of the CD3^+^TCR-β^+^ lymphocytes, the ATS group demonstrated a significantly increased percentage (6.21–19 %) (Fig. 2a) and a similar absolute number of DN T cells (Fig. 2c), suggesting that DN T cells were resistant to ATS mediated depletion.

**Fig. 3:**
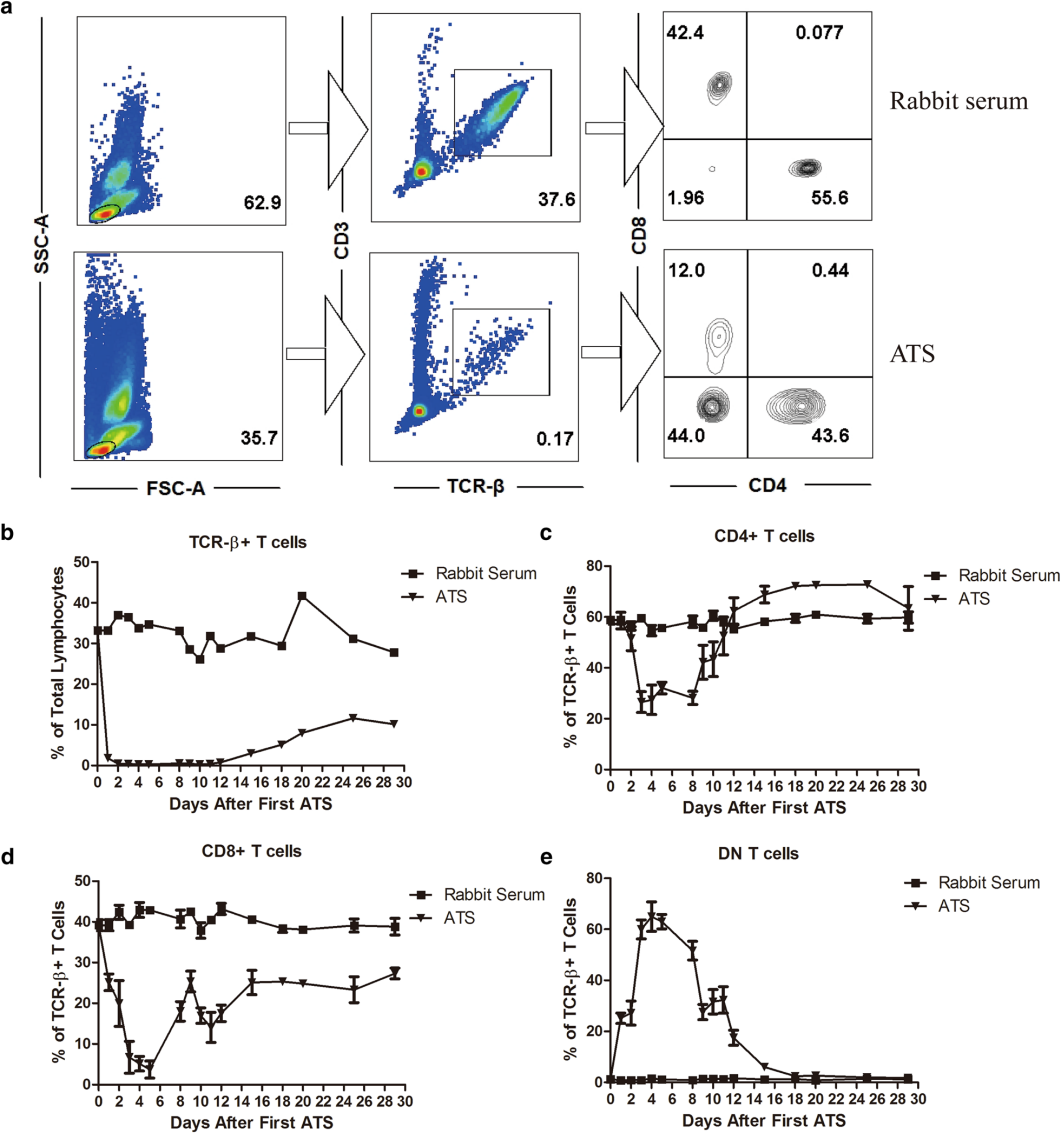
ATS treatment differentially depletes T cells from the peripheral blood in vivo. NOD mice were treated with ATS or rabbit serum and the percentage of TCR-β^+^, CD4^+^, CD8^+^ and DN T cells in the peripheral blood was examined by flow cytometry. **a** The flow cytometry results at day 9 after the first ATS treatment indicate the measurement of the percentage of different T cell subsets. The numbers in the *left panels* refer to the percentages of lymphocytes in the total PBMC, the numbers in the *middle panels* refer to the percentages of CD3^+^TCR-β^+^ cells in the total lymphocyte pool and the numbers in the *right panels* refer to the percentages of CD4^+^, CD8^+^ and DN T cells among the CD3^+^TCR-β^+^ lymphocytes. T cell subsets (based on flow cytometry analysis) from the peripheral blood were followed for 30 days after two doses of ATS. (n = 4 in each group). **b** The percentage of CD3^+^TCR-β^+^ cells in the total lymphocyte pool. **c** The percentage of CD4^+^ T cells among the CD3^+^TCR-β^+^ T cells. **d** The percentage of CD8^+^ T cells among the CD3^+^TCR-β^+^ T cells. **e** The percentage of CD4^−^CD8^−^ DN T cells among the CD3^+^TCR-β^+^ T cells

We monitored the post-ATS treatment depletion of different subsets of T cells in vivo. NOD mice were treated with two doses of ATS or rabbit serum (day 0 and 2), the percentages of different T cell subsets in the peripheral blood were examined (Fig. 3a). After treatment, we drew blood from each group (n = 4) on the day indicated in Fig. 3b–e. As shown in Fig. 3b, after ATS treatment, the TCR-β^+^ T cells in the peripheral blood were nearly depleted on day 3 (from 30 to 0.03 %), but began to recover on day 12 and were still lower on day 30 comparing with the rabbit serum group. As shown in Fig. 3c, after ATS treatment, the CD4^+^ T cell percentage of the total TCR-β^+^ T cell pool dropped to its lowest level on day 3 (from 60 to 25 %), and began to recover afterwards and returned to a normal percentage of TCR-β^+^ T cell by day 12. Compared to the rabbit serum group, the CD8^+^ T cells dropped to their lowest levels in total TCR-β^+^ T cells 5 days after ATS therapy (from 40 to 5 %). The relative percentage of CD8^+^ T cells began to recover slowly after day 5, and remained significantly lower than the control group on day 30 (Fig. 3d). Interestingly, the proportionate increase of DN T cells relative to the total TCR-β^+^ T cells (Fig. 3e) was found in ATS group on day 1 and reached its peak on day 4 post-treatment.

The data above demonstrated that ATS strongly regulates TCR-β^+^, CD4^+^ and CD8^+^ T cells both in vitro and in vivo. The most profound inhibition efficacy was observed against CD8^+^ T cells. In contrast, the in vitro and in vivo data showed that DN T cells were resistant to ATS mediated depletion.

### Combined ATS and DN T cell treatment resulted in significant reversion of new-onset autoimmune diabetes in NOD mice

DN T cells did not efficiently suppress CD8^+^ T cells in vivo, while ATS significantly depleted CD8^+^ T cells but had no strong effects on DN T cells. We therefore explored whether the combination of ATS treatment with CD4^+^ T cell converted DN T cells could reverse new onset type 1 diabetes in NOD mice. Two doses of ATS were given to diabetic NOD mice on day 0 and day 2 after diabetes onset by intraperitoneal injection. On day 7, 1 × 10^6^ ex vivo GAD65 primed DN T cells were transferred to the ATS treated mice by tail vein injection.

**Fig. 4:**
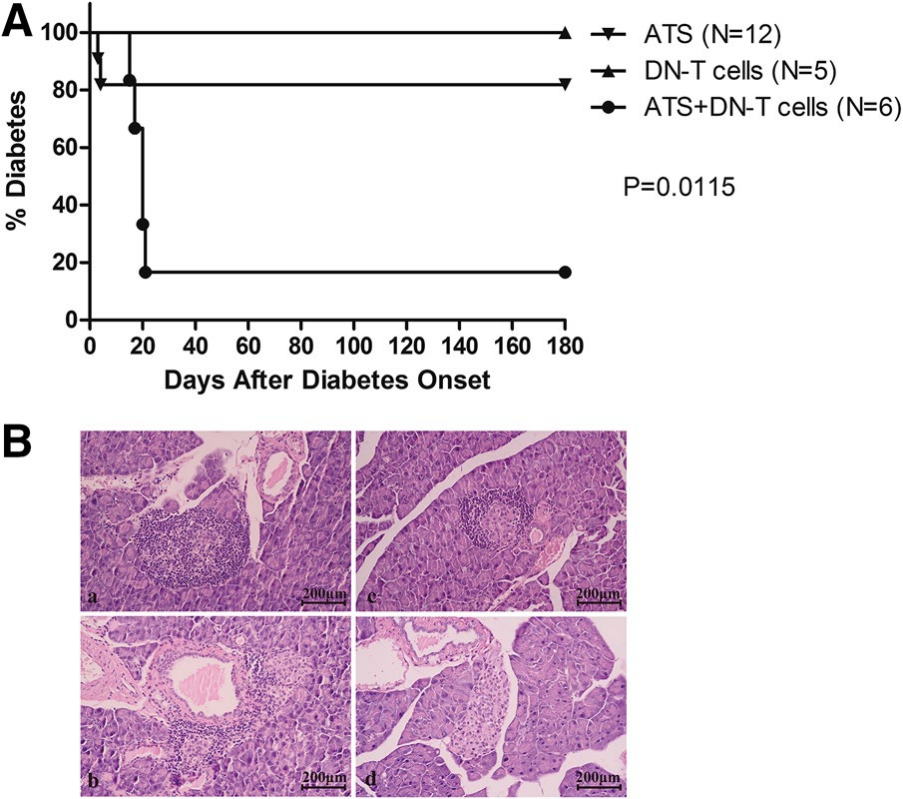
Reversion of autoimmune diabetes in new-onset type 1 diabetes NOD mice. **A** At the onset of diabetes, two doses of 50 µl ATS were given to diabetic NOD mice on day 0 and day 2 by intraperitoneal injection. On day 7, ex vivo converted GAD65 primed DN T cells (1 × 10^6^) were transferred to ATS-treated mice by tail vein injection. Mice were monitored for the diabetes reversion by measuring blood glucose levels. Statistical analysis was performed using a log-rank test. **B** Histological analysis of islets from different groups. Routine H and E staining of pancreas isolated 4 weeks after diabetes onset. Massive tissue infiltration by mononuclear cells with destruction of islets is observed in mice without treatment (*a*), with two doses of ATS treatment (*b*), DN T cells alone (*c*) and combination treatment of DN T cells plus ATS show intact islets with minimal mononuclear cell infiltration (*d*). Paraffin sections, original magnification× 400

Twenty-one days after diabetes onset, 80 % of the diabetic NOD mice (n = 6) that received the combined ATS and DN T cell treatment achieved autoimmune diabetes reversion that lasted for at least 6 months (Fig. 4A). In contrast, a single transfer of an equivalent amount of GAD65 primed DN T cells was unable to induce reversion of hyperglycemia in any recipient (n = 5). ATS induction therapy alone (n = 12) resulted in a 16 % reversion of hyperglycemia in all recipients. These data suggest that ATS induction therapy significantly promotes DN T cell efficacy in reversing autoimmune diabetes.

Histopathological changes were assessed in H and E-stained pancreas sections from NOD recipients 28 days after diabetes onset in each group (Fig. 4B). A massive mononuclear cells infiltration of the islets with loss of islet structure was observed in the no treatment group (a). Fewer mononuclear cells infiltration of the islets were observed in diabetic NOD mice that received either ATS or 1 × 10^6^ DN T cells alone (b and c). In contrast, pancreas sections from NOD mice given 1 × 10^6^ DN T cells in combination with ATS treatment (d) showed normal islet structure without obvious mononuclear cell infiltration.

### After combined ATS plus DN T cell treatment in NOD mice, the percentage of DN T cells in spleen and pancreatic draining lymph nodes (LN) is significantly higher than that in mesenteric LN

**Fig. 5:**
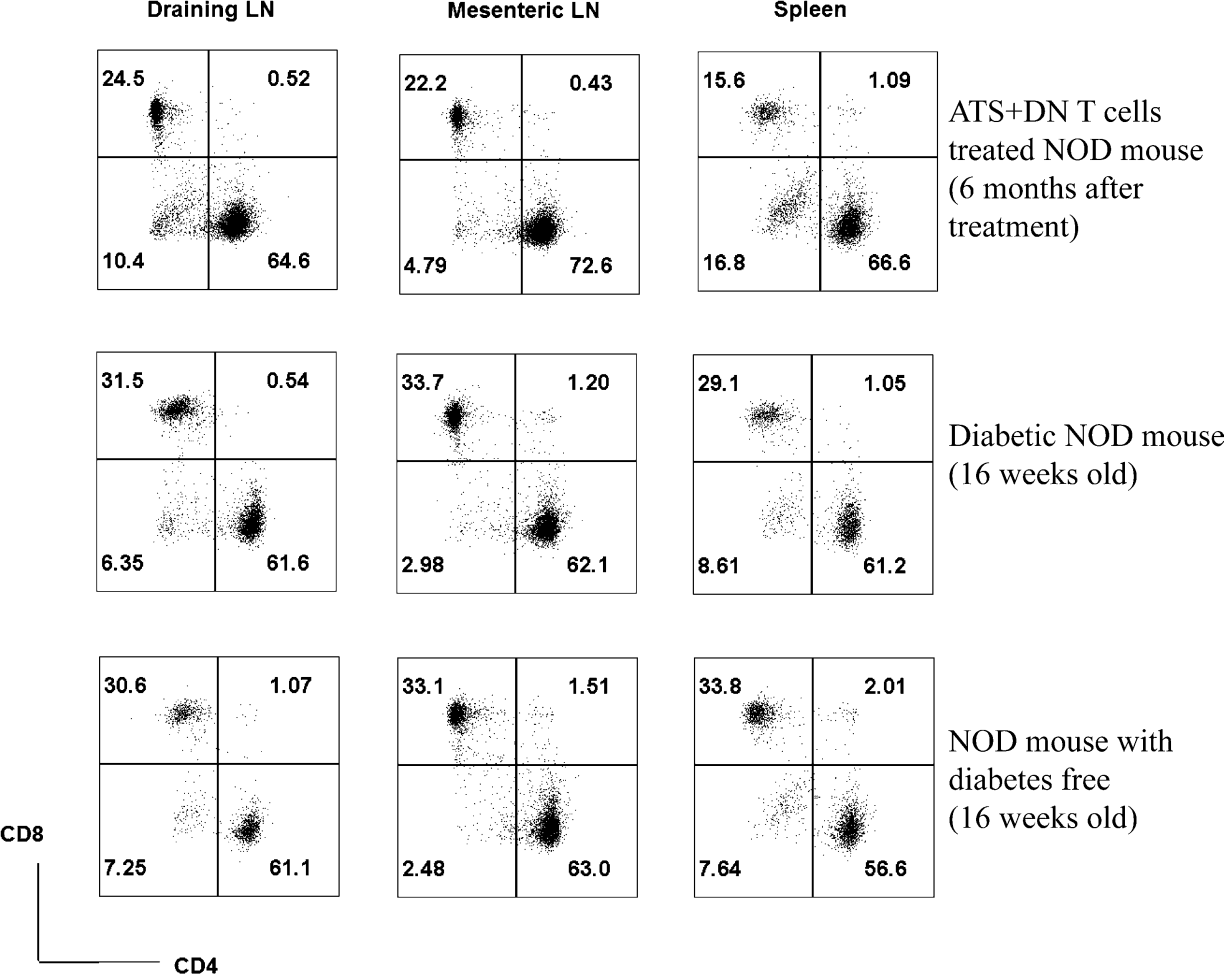
The accumulation of DN T cells in the pancreas-associated LN and the spleen as opposed to the MLN 6 months after combined ATS and DN T cell treatment. The spleen, pancreatic draining LN and mesenteric LN were harvested from different NOD mice and single cell suspensions were prepared. The percentage of CD4^+^, CD8^+^ and DN T cells in CD3+TCR-β^+^ T cells were examined by flow cytometry

To gain further understanding of the distribution of DN T cells in diabetic NOD mice cured by combined ATS plus DN T cell treatment, we investigated the percentage of DN T cells in different peripheral lymphoid tissues. As indicated in Fig. 5, the percentage of DN T cells in spleen and pancreatic draining lymph nodes (LN) is relatively higher in all groups than in mesenteric LN. Six months after combined ATS and DN T cell treatment, the percentage of DN T cells (10.4 % in draining LN, 16.8 % in the spleen) is higher in treated mice than in diabetes free control (7.25 % in draining LN, 7.64 % in the spleen) or diabetic NOD mice that received no treatment (6.35 % in draining LN, 8.61 % in the spleen). Compared with diabetes free control or diabetic NOD mice that received no treatment, in the combined treatment group, the percentage of DN T cells in draining LN (10.4 %) and the spleen (16.8 %) is significantly higher than the percentage in mesenteric LN (4.79 %). Additionally, the suppression of CD8^+^ T cells in spleen is still significant 6 months after treatment. This suggests that combined treatment leads to long-term suppression of CD8^+^ T cells.

## Discussion

Type 1 diabetes develops when the insulin-secreting beta cells are destroyed by infiltrating T cells. Autoreactive T cells, both CD4^+^ and CD8^+^ cells, have been implicated as active players in beta-cell destruction [2–5, 20]. Thus, a treatment strategy that targets both CD4^+^ as well as CD8^+^ T cells may be able to induce immunological tolerance to beta cells.

Studies reveal that DN T cells are potent T cells suppressors [15, 21, 22]. We previously demonstrated that CD4^+^ T cell converted DN T cells blocked autoimmunity and prevented diabetic onset in NOD mouse models. These effects were even greater when using islet beta cell antigen-specific DN T cells. However, reversing new-onset autoimmune diabetes was a more daunting challenge. A single transfer of 4 × 10^6^ DN T cells only slightly postponed the progression of hyperglycemia in new-onset autoimmune diabetic mice [13]. In this study, the efficacy of inhibition of DN T cells on CD8^+^ T cells was lower than on CD4^+^ T cells in vitro. Furthermore, the co-transfer of DN T cells did not protect against CD8^+^ T cell triggered skin graft rejection. These results indicate that inefficient CD8^+^ T cells suppression in vivo may be one of the reasons underlying the failure of DN T cells to control autoimmunity in new-onset diabetic NOD mice. The failure of these trials has led to efforts to more directly shift the balance from destructive a T cell response to regulatory T cell control [23].

ATG is a common immunosuppressive reagent used in allogeneic transplantation [24–26] and autoimmune disorders [27–31]. ATS is a polyclonal rabbit anti-mouse thymocyte product that is similar in action to ATG and that effectively depletes peripheral blood T cells in vivo [32]. Spontaneous diabetes in female mice was suppressed by ATS [33]. ATG therapy functions through complement mediated depletion of mature T cells, while tregs were less sensitive to ATG depletion [19]. However, researchers found that a brief course of ATG therapy does not result in the preservation of β-cell function 12 months after the treatment course in patients with new-onset type 1 diabetes [34]. Furthermore, ATG does not reverse type 1 diabetes in the acute virally induced rat insulin promoter-lymphocytic choriomeningitis virus (RIP-LCMV) model [35].

In line with previous reports, we found that ATS therapy markedly depleted TCR-β^+^, CD4^+^ and CD8^+^ T cells. Among these cell types, CD8^+^ T cells were the most sensitive to ATS depletion. We report, for the first time, that DN T cells are, like Tregs, less sensitive to ATS depletion and make up a dramatically increased percentage of the post-treatment cell population. These results could explain why ATS induction therapy resulted in a 16 % reversion of hyperglycemia in new-onset diabetic NOD mice.

Our data suggest that ATS markedly suppresses CD8^+^ T cells and selectively preserves the DN T cell population. We also demonstrated that converted DN T cells have a strong ability to regulate pathogenic CD4^+^ T cells but a lesser ability to suppress CD8^+^ T cells. We then assessed the ability of combined ATS induction and DN T cell therapy to shift the balance away from a destructive T cell response towards a DN T cell regulated response in new-onset diabetic mice. After the ATS treatment has reduced the levels of both CD4^+^ and CD8^+^ T cells, GAD65 primed DN T cells that were converted from CD4^+^ T cells in vitro were transferred 7 days after diabetes onset. In 21 days, the combined treatment achieved long term reversion of autoimmune diabetes in most of the new-onset diabetic NOD mice (80 %). However, an equivalent amount of GAD65-primed DN T cells resulted in no reversal of hyperglycemia. When used alone to treat new-onset diabetic NOD mice, ATS induction only resulted in a 16 % reversion of hyperglycemia.

Treg homing in secondary lymphoid tissues is required for the functional clustering of tregs with APCs and T cells that is necessary for the induction and maintenance of immunological tolerance [36, 37]. We first report that DN T cells preferentially migrate to the spleen and pancreatic draining LN. Six months after ATS plus DN T cell treatment, the percentage of DN T cells in mice receiving the combined treatment is higher than that observed in both diabetes free mice and diabetic NOD mice without treatment. In the combined treatment group, the percentage of DN T cells in draining LN and the spleen is significantly higher than that found in mesenteric LN. This indicates that islet specific DN T cells migrated to the pancreatic draining LN and spleen and protected the islets’ beta cells from destruction by pathogenic T cells, but that DN T cells did not migrate to other unrelated lymphoid organs. Additionally, combined treatment induced long-term suppression of CD8^+^ T cells. Taking into consideration the major role that CD8^+^ T cells play in autoimmune diabetes, the potent and long-term suppression of CD8^+^ T cells by DN T cells is likely one of the reasons for the long-term reversion of diabetes caused by the combined treatment.

## Conclusions

The combination of transient T cell depletion by ATS with adoptive transfer of ex vivo CD4^+^ T cell converted DN T cells leads to a long term reversal of new-onset diabetes in NOD mice. The improvement of outcome is due to a shift of balance from a destructive T cell response to one that favors DN T cell regulation. The results reported by this study support the concept and the potential feasibility of utilizing this novel cell-based therapeutic approach for the treatment of autoimmune type 1 diabetes.

## Abbreviations

DN: double-negative
NOD: non-obese diabetic
ATS: anti-thymocyte serum
TCR: T cell receptor
ATG: anti-thymocyte globulin
LN: lymph nodes
Tregs: regulatory T cells
